# Global quantitative indices reflecting provider process-of-care: data-base derivation

**DOI:** 10.1186/1471-2288-10-32

**Published:** 2010-04-19

**Authors:** John L Moran, Patricia J Solomon, the Adult Database Management Committee (ADMC) of the Australian and New Zealand Intensive Care Society (ANZICS)

**Affiliations:** 1 Department of Intensive Care Medicine, The Queen Elizabeth Hospital, Woodville SA 5011, Australia; 2 School of Mathematical Sciences, University of Adelaide, Adelaide SA 5000, Australia; 3 Australian and New Zealand Intensive Care Society, Carlton Victoria 3053, Australia

## Abstract

**Background:**

Controversy has attended the relationship between risk-adjusted mortality and process-of-care. There would be advantage in the establishment, at the data-base level, of global quantitative indices subsuming the diversity of process-of-care.

**Methods:**

A retrospective, cohort study of patients identified in the Australian and New Zealand Intensive Care Society Adult Patient Database, 1993-2003, at the level of geographic and ICU-level descriptors (n = 35), for both hospital survivors and non-survivors. Process-of-care indices were established by analysis of: (i) the smoothed time-hazard curve of individual patient discharge and determined by pharmaco-kinetic methods as area under the hazard-curve (AUC), reflecting the integrated experience of the discharge process, and time-to-peak-hazard (TMAX, in days), reflecting the time to maximum rate of hospital discharge; and (ii) individual patient ability to optimize output (as length-of-stay) for recorded data-base physiological inputs; estimated as a technical production-efficiency (TE, scaled [0,(maximum)1]), via the econometric technique of stochastic frontier analysis. For each descriptor, multivariate correlation-relationships between indices and summed mortality probability were determined.

**Results:**

The data-set consisted of 223129 patients from 99 ICUs with mean (SD) age and APACHE III score of 59.2(18.9) years and 52.7(30.6) respectively; 41.7% were female and 45.7% were mechanically ventilated within the first 24 hours post-admission. For survivors, AUC was maximal in rural and for-profit ICUs, whereas TMAX (≥ 7.8 days) and TE (≥ 0.74) were maximal in tertiary-ICUs. For non-survivors, AUC was maximal in tertiary-ICUs, but TMAX (≥ 4.2 days) and TE (≥ 0.69) were maximal in for-profit ICUs. Across descriptors, significant differences in indices were demonstrated (analysis-of-variance, *P *≤ 0.0001). Total explained variance, for survivors (0.89) and non-survivors (0.89), was maximized by combinations of indices demonstrating a low correlation with mortality probability.

**Conclusions:**

Global indices reflecting process of care may be formally established at the level of national patient data-bases. These indices appear orthogonal to mortality outcome.

## Background

The outcomes paradigm is now a dominant influence within medicine [1] and critical care is no exception to this movement [2]. The 1986 paper by Knaus et al [3], evaluating outcomes of a cohort of 13 intensive care units (ICU), established the notion of institutional or provider performance within the critical care discipline by way of the nexus between risk-adjusted mortality and process-of-care, the latter established through questionnaire, on-site visit and case-note review. Similar investigations were concurrently reported in the general medical literature by Dubois and colleagues [4]. A discordant debate has subsequently occurred regarding the relationship between risk-adjusted mortality and process-of-care, the latter being variously assessed [5,6]. On the one hand mortality "...is unlikely to be a sufficient statistic for quality" [7]; yet, the felicity with which process may be measured is no guarantee that "measuring ...process and reporting performance will improve outcomes" [8]. Contra-wise to the relationship of process-of-care and mortality outcome, a recent study has suggested that the "notion that hospitals with higher risk-adjusted mortality rates have poorer quality care is neither consistent nor reliable" [9]. However, there is a certain circularity in these arguments: reliance on outcome measures (mortality or length of stay [10]) is criticised from the stand point of process-of-care [11] (adherence to checklists [12]), which in turn finds its (ultimate) assessment in terms of the effect on precisely those outcomes which have been rejected in the first place.

As opposed to a piecemeal examination of single indicators or a composite-scores approach [13], there would appear to be advantage in the establishment of global quantitative indices [14,15] which would subsume the diversity of process-of-care and avoid the necessity of direct examination of the modalities of the latter [12]. We sought to establish such indices at the level of a bi-national intensive care patient data-base [16,17], the Adult Patient Database (APD) of the Australian and New Zealand Intensive Care Society (ANZICS); by analysis of:

• the hazard of patient hospital discharge, estimated using time-to-event analysis [18], as reflecting the time-course of process-of-care. The components of the time-hazard curve were determined using pharmaco-kinetic methods.

• individual patient ability to maximize output [19], in this case length of stay, for a given set of physiological inputs, the individual patient component variables of the Acute Physiology and Chronic Health Evaluation (APACHE) III severity of illness algorithm [20]. This ability was conceptualised as one of technical production efficiency ("economic" efficiency ≡ output/input, scaled [0,1] [21]), estimated by the econometric technique of stochastic frontier analysis (SFA) [22].

We also determined the degree of correlation, or independence (orthogonality), between these global process-of-care indices and mortality.

## Methods

### Data sources

As previously described [16], the ANZICS APD [17] was interrogated to define an appropriate patient set over the time period 1993-2003. In brief, physiological variables collected, in accordance with the requirements of the APACHE III algorithm [20], were the worst in the first 24 hours after ICU admission, and all first ICU admissions to a particular hospital for the period 1993-2003 were selected. Records were used only when all three components of the Glasgow Coma Score (GCS) were provided; records for which all physiologic variables were missing were excluded, and for the remaining records, missing variables were replaced with the normal range and weighted accordingly. ICU and hospital length-of-stay, initially recorded in hours, were transformed to fractional days. Patients with an ICU length of stay > 60 days and hospital length of stay > 365 days were not considered in formal analysis. Exclusions: unknown hospital vital outcome and date of discharge; patients with an ICU length of stay ≤ 4 hours; and patients aged < 16 years of age. Access to the data was granted by the ANZICS Database Management Committee in accordance with standing protocols; local hospital (The Queen Elizabeth Hospital) Ethics of Research Committee approval was waived.

### Data set-up

ICU-year units were formed by ICU-site × calendar-year interaction with a minimum patient number set at 150 to ensure estimation stability. Categorical predictors were parameterized as indicator variables with the reference level (= 0) indicated in parentheses in the following list:

• mechanical ventilation (not ventilated)

• ICU-level, as defined in the ANZICS database, as Rural, Metropolitan, Tertiary and Private (Tertiary)

• Geographical-location; that is New Zealand and the States of the Commonwealth of Australia, excluding Western Australia (New South Wales (NSW), the largest contributor). A reference population-density map is provided in Additional file 1.

• patient surgical status as post-elective surgery, post-emergency surgery and non-surgical (non-surgical)

• annual patient admission number (reflecting ICU "size"), created by the ICU-site × calendar-year interaction, was categorized at the median, the reference category being that denoting the highest number of yearly admissions

### Statistical analysis

Variables were reported as mean (SD), except where otherwise indicated. Distributional form of variables of interest was displayed using kernel density estimates and the empirical distribution and parameter 95% confidence intervals (CI) were computed via bootstrapping (1000 repetitions) [23]. In the presence of skewed distribution, median point-estimates were reported. Group differences between continuous variables were estimated using parametric or non-parametric analysis of variance (ANOVA, adjusted (Sidak) for multiple comparisons) where indicated. Stata™ (Version 10 MP, 2007; College Station, Texas) statistical software was used.

### Mortality probabilities

Hospital mortality probabilities (MP), summed over descriptors, were estimated using a two-level, patients within ICU-year units, random effects [random-intercept] logistic regression model as previously reported [16]. Descriptor standardised mortality rates (SMR) and 95%CI were estimated using the parametric approach of Rapoport et al [24].

### Hazard of discharge

The unit of analysis was patients within descriptors. For hospital survivors, length of stay of non-survivors was defined as >> the maximum length-of-stay of alive discharges, with no censoring. The hazard of hospital (or ICU) discharge was computed via time-to-event analysis with kernel density smoothing [18]. Similarly, for hospital non-survivors, length of stay of survivors was defined >> maximum length-of-stay of those dying, with no censoring [25]. Such an approach obviated the analytical problem of competing risks [26]. The smoothed hazard estimates and 95%CI, truncated at 30 days and adjusted for the mean values of the covariates age and APACHE III score for each descriptor, were output as individual data files and separately processed using standard pharmaco-kinetic techniques (the Stata™ "pk" suite of commands [27]) to estimate parameters of the "hazard" profile; in particular: peak hazard (CMAX) and time to peak hazard (TMAX), reflecting the initial intensity of and time to the maximum (rate of) patient hospital discharge, respectively; area under the hazard curve (AUC), reflecting the integrated experience of the discharge process; and the "elimination rate" (KE), the negative of the parameter estimate for a linear regression of log(time) on hazard, which determines the half-life (*t*_1/2_) of the overall hazard-profile (). The justification for this approach was that an initial (random effects) first-order compartment model [28] provided a good fit to the data (see Additional file 2). These parameter estimates were understood as global indices reflecting aspects of descriptor process-of-care.

### Technical (production) efficiency

Patients were considered as producers, seeking to avoid waste by obtaining maximised outputs from given inputs or, by minimizing input use in the production of given outputs [22]; where, in this context, "maximize" is used in the sense of optimize. The notion of productive efficiency corresponds to technical efficiency, the latter being estimated by SFA. A stochastic production frontier model may be estimated, using the Stata™ module "frontier", as a log-linear function (*f*): ; where *TE*_*i *_= exp(-*u*_*i*_) and *u*_*i *_> 0, here assumed exponentially distributed; *y*_*i *_is ICU/hospital length of stay; *x*_*ij*_s are (logged) acute physiology score and chronic health evaluation variables (age, GCS, temperature, heart rate, arterial pH, arterial PaCO_2_, creatinine, mean arterial pressure, white cell count, plasma bilirubin, plasma glucose and total (APACHE III) co-morbidity count); , *i *= 1, ..., *N *(idiosyncratic patient component). Adjustment for heteroscedasticity of the variance function of both *u *and *v *(as provided for by the Stata™ module "frontier") was undertaken in model development with a combination of appropriate patient (gender, patient surgical status), treatment (ventilation status) and provider descriptor variables (calendar year, ICU level, annual patient admission number and geographical locality). Patient efficiencies (TE, scaled [0, 1], where 1 ≡ the optimal production frontier) were summed over categorical descriptors of interest.

### Data display

The multivariate relationships (joint distribution) between the indices (MP, TE, TMAX, AUC, CMAX and KE), for survivors and non-survivors across descriptors, were displayed using biplots [29]. Biplots consist of lines, reflecting the dataset variables, and "dots" to show the observations. The length of the lines approximates the variances of the variables (the longer the line, the higher is the variance) and the cosine of the angle between the lines approximates the correlation; the closer the angle is to 90, or 270 degrees, the smaller the correlation (orthogonality); an angle of 0 or 180 degrees reflecting a correlation of 1 or -1, respectively. Variable inclusion in the biplots was adjusted to maximize the explained variance.

## Results

The data set consisted of 223129 patients from 99 ICUs over an 11 year period. Mean (SD) age and APACHE III score were 59.2 (18.9) years and 52.7 (30.6) respectively; 41.7% were female and 45.7% were mechanically ventilated within the first 24 hours post ICU-admission. Overall ICU and hospital mortalities were 10.4% and 16.1% respectively. ICU length of stay was 3.6 (5.6) (median 1.8, interquartile range 2.9 [0.9-3.8]) days and hospital length of stay was 16.4 (19.5) (median 10.1, interquartile range 14.6 [5.1-19.7]) days. Patient categorization was non-operative (55.2%), elective surgical (28.7%) and emergency surgical (16.1%). Annual patient admission number created by the ICU-site × calendar-year interaction, was categorized at the median (711 patients); this categorization was further used to create "ICU-hospital/geographical area/size" descriptors (n = 35).

### Hazard of discharge and technical efficiency

**Figure 1:**
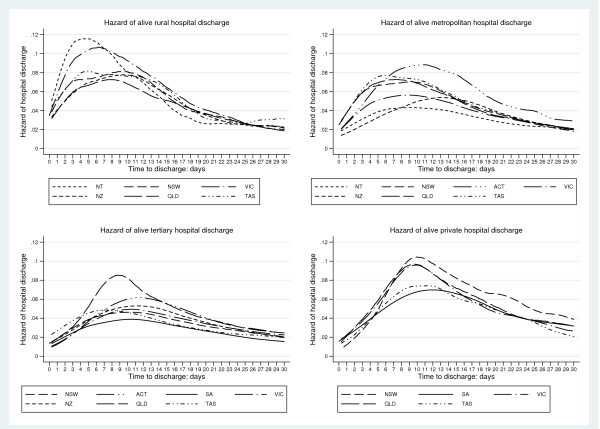
**Kernel density smoothed hazard curves of *alive-discharge *for geographic descriptors (New Zealand and States of the Commonwealth of Australia, as indicated) for rural, metropolitan, tertiary and private hospital ICUs**.

**Figure 2:**
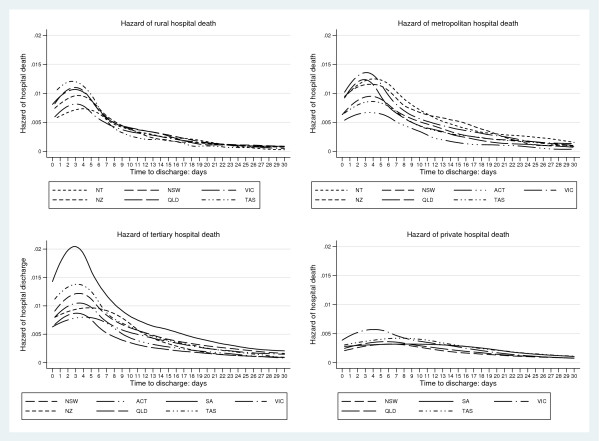
**Kernel density smoothed *mortality hazard *curves for geographic descriptors (New Zealand and States of the Commonwealth of Australia, as indicated) for rural, metropolitan, tertiary and private hospital ICUs**.

**Figure 3:**
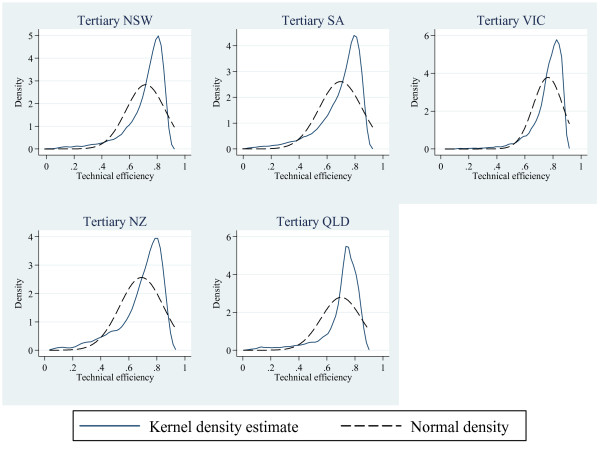
**Kernel density estimates of technical efficiencies (solid line) for various geographic descriptors, overlaid with a normal density plot**.

Hazard-of-discharge curves, for geographical areas and hospital-ICU levels, are seen for alive-discharges and those dying in Figures 1 and 2 respectively. The curves for those dying were not unexpectedly displaced to the left compared with those surviving. Initial analysis of the smoothed hazard curves used a non-linear mixed effects approach which yielded parameter estimates displaying good between-descriptor discriminative properties (Additional file 2). As this analysis required specialised software [28], standard pharmacokinetic parameters, with bootstrapped 95% CI, were calculated. Descriptor technical efficiencies (Figure 3) and mortality probabilities revealed non-normality and median point estimates were reported. Complete summaries at the level of geographical × hospital-ICU level categories × ICU "size", with standardised mortality rates, are given for both survivors and non-survivors in Tables 1 and 2 respectively.

**Table 1:** Parameter estimates and 95% CI for survivors

Descriptor	TE	AUC	KE	CMAX	TMAX
Rural NT Year admit < 711	0.590 (0.569,0.609)	2.059 (1.914,2.211)	0.042 (0.001, NA)	0.118 (0.109,0.129)	5.730 (4.628,7.827)
Rural NSW Year admit < 711	0.637 (0.628,0.644)	1.840 (1.779,1.899)	0.043 (0.023,0.062)	0.092 (0.088,0.097)	10.807 (9.907,11.150)
Rural VIC Year admit < 711	0.663 (0.655,0.672)	2.174 (2.113,2.235)	0.065 (0.044,0.085)	0.106 (0.102,0.111)	9.305 (7.205,10.505)
Rural VIC Year admit > 711	0.641 (0.614,0.658)	3.267 (3.059,3.503)	0.076 (0.018,0.147)	0.182 (0.168,0.198)	7.810 (5.711,9.953)
Rural NZ Year admit < 711	0.648 (0.632,0.658)	1.705 (1.624,1.782)	0.032 (0.008,0.059)	0.086 (0.081,0.092)	10.534 (9.358,11.153)
Rural QLD Year admit < 711	0.633 (0.622,0.642)	1.624 (1.552,1.702)	0.056 (0.030,0.084)	0.077 (0.072,0.082)	8.737 (8.138,9.635)
Rural TAS Year admit < 711	0.616 (0.577,0.652)	2.058 (1.795,2.37)	0.021 (0.001,0.088)	0.099 (0.082,0.122)	11.797 (6.425,15.394)
					
Metropolitan NT Year admit < 711	0.714 (0.708,0.719)	0.997 (0.952,1.042)	0.028 (0.014,0.044)	0.043 (0.040,0.045)	11.218 (9.343,12.936)
Metropolitan NSW Year admit < 711	0.661 (0.656,0.666)	1.539 (1.498,1.576)	0.056 (0.043,0.069)	0.074 (0.071,0.076)	10.833 (10.227,11.43)
Metropolitan NSW Year admit > 711	0.642 (0.617,0.660)	2.267 (2.160,2.39)	0.099 (0.060,0.143)	0.112 (0.105,0.120)	8.715 (6.008,10.807)
Metropolitan ACT Year admit < 711	0.729 (0.716,0.751)	2.095 (1.876,2.339)	0.050 (0.007,0.105)	0.103 (0.089,0.122)	12.012 (9.914,15.310)
Metropolitan SA Year admit > 711	0.693 (0.684,0.719)	2.574 (2.338,2.879)	0.111 (0.044,0.195)	0.145 (0.130,0.164)	7.938 (7.192,15.586)
Metropolitan VIC Year admit < 711	0.697 (0.693,0.702)	1.345 (1.315,1.379)	0.038 (0.026,0.051)	0.060 (0.058,0.062)	10.808 (9.908,11.725)
Metropolitan NZ Year admit < 711	0.742 (0.730,0.752)	1.144 (1.041,1.259)	0.054 (0.024,0.085)	0.054 (0.048,0.062)	13.868 (12.672,16.257)
Metropolitan QLD Year admit < 711	0.650 (0.645,0.656)	1.626 (1.571,1.680)	0.047 (0.027,0.066)	0.076 (0.073,0.080)	9.062 (7.866,10.259)
Metropolitan QLD Year admit > 711	0.700 (0.692,0.704)	1.621 (1.549,1.709)	0.046 (0.018,0.074)	0.077 (0.073,0.083)	9.344 (7.847,10.840)
Metropolitan TAS Year admit < 711	0.668 (0.658,0.680)	1.697 (1.601,1.800)	0.057 (0.022,0.091)	0.082 (0.075,0.089)	9.980 (6.693,11.474)
					
Tertiary NSW Year admit < 711	0.766 (0.764,0.767)	0.949 (0.932,0.967)	0.029 (0.022,0.036)	0.041 (0.040,0.042)	12.331 (12.004,12.654)
Tertiary NSW Year admit > 711	0.739 (0.735,0.742)	1.312 (1.270,1.345)	0.042 (0.032,0.054)	0.059 (0.057,0.061)	9.302 (8.403,9.614)
Tertiary ACT Year admit < 711	0.799 (0.797,0.801)	0.809 (0.772,0.847)	0.002 (0.001,0.003)	0.030 (0.028,0.031)	23.805 (22.625,24.985)
Tertiary ACT Year admit > 711	0.807 (0.805,0.810)	1.331 (1.280,1.389)	0.039 (0.021,0.057)	0.066 (0.063,0.070)	11.508 (10.911,11.957)
Tertiary SA Year admit < 711	0.738 (0.734,0.742)	1.034 (1.006,1.061)	0.050 (0.037,0.063)	0.048 (0.046,0.050)	11.203 (10.31,11.802)
Tertiary SA Year admit > 711	0.750 (0.746,0.754)	0.691 (0.670,0.713)	0.042 (0.032,0.053)	0.029 (0.028,0.031)	11.426 (10.827,12.627)
Tertiary VIC Year admit < 711	0.788 (0.784,0.791)	1.042 (1.007,1.076)	0.032 (0.020,0.042)	0.048 (0.046,0.050)	12.262 (11.71,12.854)
Tertiary VIC Year admit > 711	0.790 (0.789,0.792)	1.081 (1.063,1.100)	0.037 (0.029,0.045)	0.049 (0.048,0.050)	10.581 (10.531,11.429)
Tertiary NZ Year admit < 711	0.734 (0.728,0.741)	1.151 (1.115,1.189)	0.046 (0.037,0.057)	0.052 (0.050,0.055)	12.503 (11.613,13.114)
Tertiary QLD Year admit < 711	0.694 (0.687,0.700)	1.331 (1.284,1.381)	0.039 (0.019,0.057)	0.062 (0.058,0.065)	9.429 (8.863,10.622)
Tertiary QLD Year admit > 711	0.746 (0.744,0.747)	1.631 (1.596,1.668)	0.038 (0.025,0.053)	0.095 (0.092,0.098)	9.308 (9.008,9.324)
Tertiary TAS Year admit < 711	0.698 (0.685,0.710)	1.067 (1.014,1.129)	0.024 (0.008,0.042)	0.048 (0.045,0.051)	8.460 (7.531,9.628)
					
Private NSW Year admit < 711	0.748 (0.744,0.753)	2.166 (2.081,2.254)	0.045 (0.022,0.066)	0.109 (0.104,0.115)	11.107 (10.421,11.402)
Private SA Year admit < 711	0.756 (0.752,0.760)	1.693 (1.605,1.783)	0.033 (0.020,0.044)	0.075 (0.069,0.080)	13.775 (13.185,14.721)
Private VIC Year admit < 711	0.761 (0.756,0.765)	1.890 (1.844,1.934)	0.045 (0.032,0.061)	0.102 (0.099,0.106)	10.222 (9.968,10.522)
Private QLD Year admit < 711	0.730 (0.727,0.733)	1.998 (1.935,2.062)	0.039 (0.022,0.056)	0.103 (0.099,0.108)	10.248 (9.855,10.750)
Private QLD Year admit > 711	0.744 (0.740,0.748)	1.735 (1.628,1.849)	0.034 (0.007,0.064)	0.091 (0.084,0.100)	10.771 (10.422,11.609)
Private TAS Year admit < 711	0.716 (0.674,0.760)	1.622 (1.403,1.860)	0.078 (0.029,0.144)	0.079 (0.067,0.094)	12.031 (10.534,18.320)

Descriptor; ICU-hospital level, geographical locality and yearly admission number (< 711 >). N/A; not computed. TE; technical efficiency.

AUC; area under hazard curve. CMAX; peak hazard. TMAX; time (days) to peak hazard).

KE; "elimination rate". 95%CI; computed via bootstrap (1000 samples)

**Table 2:** Parameter estimates and 95% CI for non-survivors

Descriptor	MP	SMR	TE	AUC	KE	CMAX	TMAX
Rural NT Year admit < 711	0.027 (0.023,0.030)	0.945 (0.846,1.044)	0.638 (0.616,0.677)	0.092 (0.078,0.106)	0.181 (0.086,0.338)	0.007 (0.005,0.009)	4.171 (3.339,4.820)
Rural NSW Year admit < 711	0.037 (0.035,0.039)	1.038 (0.992,1.083)	0.612 (0.598,0.627)	0.120 (0.113,0.128)	0.051 (0.012,0.098)	0.010 (0.009,0.011)	3.019 (2.719,3.320)
Rural VIC Year admit < 711	0.033 (0.032,0.035)	0.992 (0.948,1.036)	0.632 (0.621,0.645)	0.102 (0.096,0.108)	0.120 (0.077,0.160)	0.008 (0.007,0.009)	3.328 (3.028,3.628)
Rural VIC Year admit > 711	0.020 (0.019,0.022)	1.006 (0.878,1.135)	0.571 (0.529,0.614)	0.058 (0.047,0.068)	0.035 (0.001,0.135)	0.006 (0.004,0.008)	3.024 (2.425,3.624)
Rural NZ Year admit < 711	0.036 (0.033,0.040)	0.988 (0.919,1.058)	0.558 (0.537,0.585)	0.105 (0.095,0.116)	0.063 (0.008,0.141)	0.009 (0.008,0.011)	3.374 (3.059,3.960)
Rural QLD Year admit < 711	0.041 (0.038,0.045)	0.993 (0.935,1.052)	0.601 (0.587,0.621)	0.120 (0.107,0.131)	0.034 (0.003,0.089)	0.100 (0.009,0.011)	3.006 (2.706,3.356)
Rural TAS Year admit < 711	0.041 (0.032,0.051)	1.000 (0.786,1.214)	0.484 (0.376,0.549)	NA (NA, NA)	NA (NA, NA)	NA (NA, NA)	NA (NA, NA)
							
Metropolitan NT Year admit < 711	0.086 (0.079,0.093)	1.017 (0.965,1.070)	0.715 (0.707,0.720)	0.177 (0.163,0.191)	0.060 (0.028,0.097)	0.012 (0.011,0.014)	4.515 (3.915,4.883)
Metropolitan NSW Year admit < 711	0.062 (0.060,0.065)	0.986 (0.953,1.018)	0.700 (0.694,0.706)	0.137 (0.130,0.144)	0.062 (0.037,0.087)	0.009 (0.009,0.010)	4.212 (3.912,4.535)
Metropolitan NSW Year admit > 711	0.041 (0.039,0.043)	1.003 (0.919,1.086)	0.647 (0.623,0.679)	0.093 (0.082,0.103)	0.092 (0.029,0.174)	0.007 (0.006,0.008)	3.909 (3.309,4.568)
Metropolitan ACT Year admit < 711	0.037 (0.033,0.043)	1.000 (0.785,1.215)	NA (NA, NA)	0.079 (0.056,0.103)	0.118 (0.008,0.121)	0.006 (0.003,0.009)	4.263 (2.766,5.854)
Metropolitan SA Year admit > 711	0.016 (0.014,0.018)	0.909 (0.694,1.124)	0.670 (0.631,0.700)	0.033 (0.021,0.049)	0.046 (0.012,0.128)	0.001 (0.001,0.002)	11.013 (7.486,16.966)
Metropolitan VIC Year admit < 711	0.083 (0.080,0.087)	0.994 (0.964,1.025)	0.680 (0.672,0.684)	0.162 (0.154,0.169)	0.076 (0.049,0.103)	0.013 (0.012,0.014)	3.315 (3.016,3.615)
Metropolitan NZ Year admit < 711	0.088 (0.077,0.100)	1.056 (0.933,1.179)	0.635 (0.612,0.662)	0.164 (0.136,0.189)	0.113 (0.026,0.216)	0.011 (0.008,0.013)	4.230 (3.031,5.428)
Metropolitan QLD Year admit < 711	0.048 (0.044,0.051)	1.026 (0.979,1.073)	0.651 (0.645,0.665)	0.127 (0.119,0.136)	0.080 (0.032,0.130)	0.011 (0.010,0.012)	3.037 (3.028,3.337)
Metropolitan QLD Year admit > 711	0.044 (0.039,0.049)	1.013 (0.945,1.082)	0.609 (0.591,0.626)	0.126 (0.114,0.138)	0.053 (0.006,0.114)	0.011 (0.010,0.013)	3.028 (2.728,3.628)
Metropolitan TAS Year admit < 711	0.046 (0.043,0.050)	1.030 (0.939,1.120)	0.604 (0.585,0.633)	0.116 (0.100,0.131)	0.081 (0.028,0.143)	0.008 (0.007,0.010)	3.984 (3.386,4.859)
							
Tertiary NSW Year admit < 711	0.111 (0.107,0.114)	1.007 (0.988,1.027)	0.720 (0.716,0.723)	0.181 (0.173,0.188)	0.062 (0.049,0.077)	0.012 (0.011,0.012)	4.207 (3.907,4.226)
Tertiary NSW Year admit > 711	0.054 (0.053,0.056)	0.988 (0.96,1.016)	0.676 (0.670,0.684)	0.142 (0.133,0.150)	0.053 (0.028,0.077)	0.010 (0.010,0.011)	3.319 (3.306,3.628)
Tertiary ACT Year admit < 711	0.060 (0.055,0.064)	1.015 (0.944,1.086)	0.640 (0.629,0.651)	0.093 (0.084,0.102)	0.086 (0.072,0.101)	0.004 (0.004,0.005)	7.875 (7.109,8.760)
Tertiary ACT Year admit > 711	0.052 (0.049,0.056)	0.991 (0.929,1.054)	0.591 (0.562,0.616)	0.141 (0.106,0.158)	0.085 (0.047,0.132)	0.008 (0.007,0.009)	3.601 (3.001,4.208)
Tertiary SA Year admit < 711	0.132 (0.127,0.138)	0.984 (0.954,1.014)	0.685 (0.680,0.689)	0.228 (0.179,0.243)	0.056 (0.033,0.080)	0.013 (0.012,0.014)	3.901 (3.601,3.934)
Tertiary SA Year admit > 711	0.228 (0.215,0.238)	1.019 (0.992,1.046)	0.626 (0.619,0.633)	0.310 (0.295,0.323)	0.073 (0.043,0.106)	0.025 (0.023,0.027)	3.008 (2.708,3.308)
Tertiary VIC Year admit < 711	0.094 (0.088,0.100)	1.001 (0.958,1.044)	0.706 (0.701,0.712)	0.150 (0.140,0.159)	0.076 (0.054,0.102)	0.009 (0.008,0.010)	5.152 (4.578,5.565)
Tertiary VIC Year admit > 711	0.077 (0.075,0.08)	1.008 (0.983,1.032)	0.669 (0.664,0.674)	0.152 (0.135,0.159)	0.047 (0.028,0.066)	0.010 (0.009,0.010)	3.602 (3.602,3.909)
Tertiary NZ Year admit < 711	0.079 (0.075,0.084)	1.001 (0.958,1.043)	0.633 (0.623,0.642)	0.135 (0.125,0.146)	0.071 (0.048,0.093)	0.009 (0.008,0.010)	5.129 (4.544,5.728)
Tertiary QLD Year admit < 711	0.059 (0.053,0.063)	1.011 (0.962,1.059)	0.695 (0.686,0.704)	0.140 (0.130,0.150)	0.036 (0.006,0.070)	0.010 (0.009,0.012)	3.96 (3.652,4.272)
Tertiary QLD Year admit > 711	0.030 (0.029,0.031)	0.976 (0.934,1.017)	0.644 (0.634,0.651)	0.084 (0.079,0.088)	0.068 (0.038,0.100)	0.007 (0.006,0.007)	3.329 (3.029,3.629)
Tertiary TAS Year admit < 711	0.101 (0.094,0.111)	0.989 (0.925,1.053)	0.607 (0.593,0.630)	0.17 (0.154,0.187)	0.098 (0.045,0.158)	0.013 (0.011,0.015)	3.393 (3.009,3.944)
							
Private NSW Year admit < 711	0.024 (0.023,0.026)	0.992 (0.909,1.075)	0.743 (0.727,0.761)	0.056 (0.050,0.063)	0.053 (0.025,0.089)	0.003 (0.003,0.004)	6.648 (5.810,7.580)
Private SA Year admit < 711	0.039 (0.035,0.043)	1.000 (0.909,1.091)	0.695 (0.680,0.707)	0.074 (0.065,0.085)	0.068 (0.049,0.088)	0.003 (0.003,0.004)	8.146 (6.649,10.241)
Private VIC Year admit < 711	0.027 (0.026,0.028)	1.007 (0.963,1.050)	0.722 (0.715,0.729)	0.089 (0.081,0.096)	0.087 (0.054,0.119)	0.005 (0.005,0.006)	4.212 (3.903,4.812)
Private QLD Year admit < 711	0.022 (0.021,0.024)	1.013 (0.945,1.080)	0.723 (0.716,0.731)	0.058 (0.053,0.064)	0.052 (0.025,0.083)	0.003 (0.003,0.004)	6.410 (5.440,7.292)
Private QLD Year admit > 711	0.034 (0.031,0.037)	1.006 (0.894,1.117)	0.691 (0.670,0.706)	0.083 (0.071,0.096)	0.076 (0.043,0.113)	0.004 (0.003,0.005)	7.264 (6.068,8.461)
Private TAS Year admit < 711	0.013 (0.010,0.016)	0.979 (0.796,1.162)	0.698 (0.650,0.737)	0.083 (0.057,0.109)	0.063 (0.016,0.322)	0.004 (0.002,0.006)	7.862 (5.448,12.050)

Descriptor; ICU-hospital level, geographical locality and yearly admission number (< 711 | > 711).

MP; mortality probability (median, 95% BCa bootstrapped intervals).

N/A; not computed. TE; technical efficiency. AUC; area under the hazard curve. CMAX; peak hazard. TMAX; time (days) to peak hazard).

KE; "elimination rate". 95%CI; computed via bootstrap (1000 samples). SMR; standardised mortality ratio (parametric 95%CI)

Summary statistics for process-of-care indices and mortality probabilities, for both survivors and non-survivors, are displayed for descriptors ICU "size" (> 711, < 711 admissions per year); ICU level by "size" and geographical-location by "size" in Tables 3 and 4. For both survivors and non-survivors, indices demonstrated statistically significant differences between the individual categories of various descriptors: ICU "size" (> 711, < 711 admissions per year); ICU level; ICU level by "size"; geographical-location by "size"; and geographical × hospital-ICU level categories × ICU "size" (ANOVA, *P *≤ 0.0001); albeit these differences reflected the large size of both the initial data-set and the empirical distributions of the indices (1000 bootstrap samples). *Maximal values of indices *were seen (Tables 3 and 4; ANOVA, P < 0.001 compared with all other ICU levels) for:

the descriptor ICU level by "size":

1. for survivors, at the rural and private level (both < 711 and >711 yearly admissions) for AUC; at tertiary levels (both < 711 and >711 yearly admissions) for TMAX and TE; at rural (>711 yearly admissions) and metropolitan (<711 yearly admissions) levels for KE; at rural and private levels for CMAX.

2. for non-survivors, at the tertiary level (both < 711 and >711 yearly admissions) for AUC; at the private level for TMAX; at rural (<711 yearly admissions) and metropolitan (<711 yearly admissions) levels for KE; at the tertiary levels (both < 711 and >711 yearly admissions) for CMAX

the descriptor ICU "size":

1. for survivors: for ICUs with > 711 admissions per year for AUC and TE; for ICUs with < 711 admissions per year for TMAX

2. for non-survivors: for ICUs with > 711 admissions per year for AUC; for ICUs with < 711 admissions per year for TMAX and TE.

**Table 3:** Summary statistics for survivors for various descriptors

Descriptor	AUC	CMAX	TMAX	KE	TE*
Admit < 711	1.587 (0.464)	0.078 (0.028)	11.214 (3.255)	0.045 (0.025)	0.726 (0.182)
Admit > 711	1.657 (0.720)	0.084 (0.043)	9.800 (1.458)	0.052 (0.026)	0.759 (0.127)
					
Rural <711	1.912 (0.215)	0.097 (0.014)	9.389 (2.342)	0.045 (0.020)	0.640 (0.239)
Rural >711	3.268 (0.112)	0.182 (0.007)	7.697 (1.409)	0.079 (0.033)	0.641 (0.200)
Metropolitan <711	1.701 (0.502)	0.083 (0.031)	10.658 (2.050)	0.061 (0.033)	0.680 (0.212)
Metropolitan >711	1.623 (0.039)	0.078 (0.003)	9.207 (0.847)	0.046 (0.014)	0.700 (0.175)
Tertiary <711	1.055 (0.152)	0.047 (0.009)	12.907 (4.690)	0.032 (0.016)	0.760 (0.150)
Tertiary >711	1.209 (0.313)	0.060 (0.022)	10.401 (1.127)	0.040 (0.007)	0.764 (0.122)
Private < 711	1.859 (0.201)	0.094 (0.014)	11.473 (1.607)	0.047 (0.022)	0.744 (0.111)
					
NT <711	1.531 (0.536)	0.081 (0.038)	8.491 (2.843)	0.027 (0.013)	0.694 (0.224)
NSW < 711	1.624 (0.499)	0.079 (0.025)	11.206 (0.787)	0.043 (0.013)	0.734 (0.175)
NSW >711	1.790 (0.482)	0.086 (0.027)	8.893 (0.757)	0.071 (0.032)	0.733 (0.170)
VIC <711	1.613 (0.445)	0.079 (0.026)	10.550 (1.408)	0.045 (0.015)	0.728 (0.186)
VIC >711	2.174 (1.096)	0.116 (0.067)	9.262 (1.869)	0.058 (0.031)	0.788 (0.111)
SA <711	1.771 (0.640)	0.089 (0.041)	11.206 (2.5010	0.065 (0.042)	0.744 (0.157)
SA >711	0.691 (0.011)	0.029 (0.001)	11.504 (0.493)	0.042 (0.005)	0.750 (0.165)
NZ <711	1.334 (0.266)	0.064 (0.016)	12.333 (1.627)	0.044 (0.015)	0.713 (0.196)
ACT <711	1.455 (0.652)	0.067 (0.037)	18.098 (5.865)	0.026 (0.031)	0.796 (0.102)
ACT >711	1.332 (0.027)	0.066 (0.002)	11.420 (0.279)	0.039 (0.001)	0.807(0.087)
QLD <711	1.645 (0.239)	0.080 (0.015)	9.477 (0.705)	0.045 (0.013)	0.693 (0.193)
QLD >711	1.654 (0.061)	0.088 (0.008)	9.636 (0.893)	0.040 (0.013)	0.741 (0.100)
TAS <711	1.613 (0.370)	0.077 (0.020)	10.708 (2.414)	0.051 (0.031)	0.682 (0.211)

Statistics as mean(SD); * Median and IQR.

States of the Commonwealth of Australia (NT: Northern territory; NSW: New South Wales; VIC: Victoria;

SA: South Australia; ACT: Australian Capital Territory; QLD: Queensland; TAS: Tasmania) and New Zealand (NZ)

Descriptor; Admit <711, > 711: yearly admission number (< 711 >); TE; technical efficiency. AUC; area under hazard curve.

CMAX; peak hazard. TMAX; time (days) to peak hazard). KE; "elimination rate".

**Table 4:** Summary statistics for non-survivors for various descriptors

Descriptor	MP*	AUC	CMAX	TMAX	KE	TE*
Admit < 711	0.058 (0.199)	0.119 (0.044)	0.008 (0.003)	4.937 (2.117)	0.079 (0.048)	0.687 (0.137)
Admit > 711	0.059 (0.204)	0.132 (0.071)	0.010 (0.006)	3.677 (1.113)	0.067 (0.028)	0.651 (0.159)
						
Rural <711	0.0353 (0.131)	0.108 (0.012)	0.009 (0.005)	3.414 (0.459)	0.095 (0.068)	0.612 (0.185)
Rural >711	0.020 (0.054)	0.058 (0.005)	0.006 (0.001)	3.062 (0.316)	0.044 (0.036)	0.572 (0.204)
Metropolitan <711	0.060 (0.198)	0.012 (0.044)	0.009 (0.003)	4.763 (2.482)	0.085 (0.053)	0.683 (0.132)
Metropolitan >711	0.044 (0.166)	0.126 (0.006)	0.011 (0.001)	3.068 (0.241)	0.053 (0.028)	0.609 (0.156)
Tertiary <711	0.099 (0.274)	0.155 (0.037)	0.010 (0.003)	4.814 (1.404)	0.070 (0.024)	0.695 (0.129)
Tertiary >711	0.066 (0.226)	0.164 (0.077)	0.012 (0.007)	3.398 (0.300)	0.065 (0.021)	0.652 (0.159)
Private < 711	0.026 (0.080)	0.073 (0.014)	0.004 (0.001)	6.811 (1.625)	0.067 (0.023)	0.718 (0.107)
						
NT <711	0.059 (0.224)	0.134 (0.043)	0.009 (0.003)	4.271 (0.362)	0.126 (0.081)	0.707 (0.116)
NSW < 711	0.069 (0.229)	0.124 (0.045)	0.009 (0.003)	4.524 (1.310)	0.058 (0.016)	0.708 (0.116)
NSW >711	0.051 (0.185)	0.117 (0.025)	0.009 (0.002)	3.683 (0.344)	0.0732 (0.034)	0.674 (0.131)
VIC <711	0.048 (0.166)	0.125 (0.031)	0.009 (0.003)	0.031 (0.794)	0.090 (0.024)	0.686 (0.131)
VIC >711	0.068 (0.200)	0.103 (0.046)	0.008 (0.002)	3.493 (0.372)	0.046 (0.022)	0.667 (0.153)
SA <711	0.106 (0.283)	0.108 (0.079)	0.006 (0.005)	7.762 (3.364)	0.060 (0.022)	0.686 (0.139)
SA >711	0.191 (0.428)	0.331 (0.007)	0.025 (0.001)	3.001 (0.110)	0.073 (0.016)	0.626 (0.170)
NZ <711	0.063 (0.210)	0.134 (0.026)	0.010 (0.001)	4.293 (0.777)	0.084 (0.041)	0.622 (0.164)
ACT <711	0.056 (0.153)	0.086 (0.012)	0.005 (0.001)	6.029 (1.947)	0.115 (0.084)	0.640 (0.150)
ACT >711	0.052 (0.174)	0.135 (0.017)	0.008 (0.001)	3.628 (0.309)	0.086 (0.021)	0.591 (0.202)
QLD <711	0.037 (0.139)	0.111 (0.032)	0.009 (0.003)	4.167 (1.401)	0.053 (0.027)	0.675 (0.148)
QLD >711	0.032 (0.112)	0.010 (0.021)	0.007 (0.003)	4.193 (1.813)	0.065 (0.024)	0.640 (0.156)
TAS <711	0.063 (0.211)	0.123 (0.037)	0.008 (0.004)	5.259 (2.319)	0.083 (0.033)	0.607 (0.204)

Statistics as mean(SD); * Median and IQR.

States of the Commonwealth of Australia (NT: Northern territory; NSW: New South Wales; VIC: Victoria; SA: South Australia; ACT: Australian Capital Territory; QLD: Queensland; TAS: Tasmania) and New Zealand (NZ)

Descriptor; Admit <711, > 711: yearly admission number (< 711 >); MP; mortality probability. TE; technical efficiency. AUC; area under hazard curve. CMAX; peak hazard. TMAX; time (days) to peak hazard. KE; "elimination rate".

### Multivariate relationships between indices

**Figure 4:**
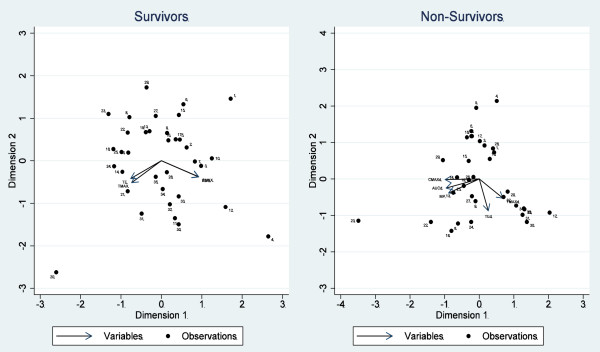
**Biplots for survivors and non-survivors demonstrating the multivariate relationships between global performance indices**. CMAX: peak hazard; TMAX: time to peak hazard; AUC: area under the hazard curve; KE: "elimination rate", TE: technical efficiency; MP: median mortality probability.

The relationships between the global indices are demonstrated in Figure 4. For survivors (left panel), the total explained variance was 0.886, and the relationships appeared discrete: both technical efficiency (TE) and time to maximal hazard (TMAX) were coincident and tended to be orthogonal to indices reflecting the maximum hazard of alive (hospital) discharge (CMAX) and the total discharge experience of the patient (AUC). When included in the plot, mortality probability (and SMR) was, not surprisingly, directionally opposite to that of AUC and did not increase the explained variance. For non-survivors (right panel), the total explained variance was 0.892. AUC and mortality probability were almost coincident and directionally equivalent to CMAX, but orthogonal to both TMAX and TE. The SMR and mortality probability were coincident (not shown). The "elimination rate" (KE) added no increment to the total explained variation for survivors or non-survivors.

## Discussion

We have proffered a number of global indices, their uncertainty estimates and multivariate relationships, as reflecting the integrated discharge process for both survivors and non-survivors at the level of various data-base descriptors. For each of the indices, we were able to establish formal statistical difference between, and characteristic clustering within, database descriptor categories for both survivors and non-survivors. These indices would appear to be both internally consistent and plausible proxies for the underlying process(es)-of-care which, in concert with patient characteristics, determine the shape of the particular descriptor hazard of discharge.

Our motivating concepts were two-fold:

1. that of "conditional length of stay", introduced by Silber and colleagues, as the length of stay after a stay is prolonged, reflecting both patient complications and/or co-morbid medical conditions, and provider ability to manage complicated cases [30,31]. As suggested by Silber et al, and based upon empirical analyses: "By studying the CLOS, one can determine when the rate of hospital discharge begins to diminish-without the need to directly observe complications. Policy makers looking for an objective outcome measure may find that CLOS aids in the analysis of a hospital's management of complicated patients..." [31]. The analytic focus was restricted to the prolongation point or day, as estimated by the Hollander and Proschan statistic [32]. Our development of this approach involved modelling the hazard-profile as a "concentration" curve with the estimation of standard pharmaco-kinetic based parameters which would allow a more complete description of the hazard-time curve, in the sense of modelling underlying processes, and allowing formal statistical comparisons.

2. the notion of efficiency. In an innovative study of patients with severe head trauma, Nathanson et al [19] used data envelopment analysis (DEA) to calculate individual patient "efficiency " scores ([0 to 1]) based upon the ability to maximize output (in this case, cerebral perfusion pressure) for a given set of physiological inputs. Patients with high efficiency scores were found to have an improved functional outcome on ICU discharge. At the individual ICU level, Junoy [33] used DEA to establish an efficiency frontier across the bivariate relationship between severity adjusted survival (effectiveness or output) and length of stay (resources or input) and to compute technical efficiency of "output quality". Length of stay, raw, adjusted or observed minus expected, has been used as an indicator of ICU/hospital performance, from both an economic [34] and quality [35] perspective. Traditionally, length of stay has been estimated by ordinary least squares regression (or its variants) by maximising the mean expectation; the residual (difference between observed and predicted) being interpreted, in the current context, as arising from inefficiency. We applied the concept of "efficiency" to individual patient length of stay to model technical production efficiency using a parametric SFA approach. The latter separates the residual into an inefficiency component (*u*_*i*_, positive departures from the (best practice) production frontier) and all other sources of model error (*v*_*i*_), such as random shocks and measurement error [15,36]. To this extent it is less sensitive to outliers than DEA, a deterministic non-parametric technique, assuming no measurement error and requiring a more rigid best-practice production frontier based upon a small subset of efficient peers [36].

That these indices captured aspects of descriptor process-of-care was suggested by the analysis of maximum values. For instance, tertiary level ICUs where more complex and severely ill patients were located [16] and human and material resources were presumably greatest, demonstrated maximum TE for survivors and AUC for non-survivors; whereas, at the rural and for-profit ICU levels, where such patient and resource conditions did not necessarily obtain, AUC was maximal for survivors. With respect to ICU "size", maximal TE was located in tertiary ICUs (> 711 yearly admissions) for survivors; but of interest, for non-survivors, was located in Private ICUs (< 711 admissions per year). These "size" effects must be interpreted against the favourable effect of ICU "size" < 711 yearly admissions on hospital mortality (OR 0.84, *P *< 0.0001 compared with > 711 yearly admissions) adduced in a previous analysis of the same data-base [16]. The regional-geographic differences in these indices (Tables 1, 2, 3, 4, above) presumably reflect both the particular nature of our data-base and determinants such as the distribution of human [37,38] and non-human resources [39] and socio-economic factors.

Analysis of the joint distribution of the global process-of-care indices revealed a weak correlation with, or orthogonality to, mortality outcome (SMR and/or mortality probability). This would appear to be the first formal demonstration of such a (lack-of) relationship and is consistent with the literature reviews of Thomas and Hofer [40] and Pitches et al [9], although both reviews noted considerable and possibly confounding between-study heterogeneity in recorded process-of-care and mortality risk-adjustment measures. The low sensitivity of individual process-of-care measures has also been previously noted [41]. This being said, any such formal "independence" of these measures would have important implications for both health policy and the design and interpretation of trials assessing process-of-care interventions [42].

### Critique of methodology

Our postulates are predicated upon the utility of both the pharmaco-kinetic and efficiency analyses. The former would appear to have basis in the good fit of a first-order compartment model; this approach may be formally extended to embrace non-linear mixed effects modelling [28]. Controversy has attended the appropriate form of efficiency analysis [43] and the use of either DEA or SFA; much of this criticism is context dependent; for instance, the efficiency of public services where multiple outputs occur. Our use of SFA was motivated by modelling flexibility and potential extensions to accommodate random effects and different distributions for the inefficiency component [44,45].

Our analysis, at the level of a bi-national database, lacks the empirical grounding of the investigations of Silber and co-workers [30,31] mentioned above and would require such validation. However, at the analytic level, these indices appeared both consistent and intuitively reasonable. In the interests of parsimony the primary descriptors were geographical and hospital-ICU level categories and the data-set was considered as a single cross-sectional unit; that is no analysis by calendar year was undertaken, although this ensured estimation stability. Similarly, we considered only hospital outcomes and truncated analysis time to 30 days, the latter again for estimation stability in the output files from the smoothed hazard estimates. However, our approach is rich with possibilities for various extensions to the individual ICU level and ICU hazard-of-discharge, longitudinal analysis [46], and obviously, different non-ICU patient cohorts.

## Conclusions

Global indices reflecting process of care may be formally established at the level of national patient data-bases, thus allowing comparisons between providers/descriptors. These indices appear orthogonal to mortality outcome; such a relationship would have implications for health care policy and the design and interpretation of trials assessing process-of-care interventions.

## Competing interests

The authors declare that they have no competing interests.

## Authors' contributions

The study was conceived, designed, (data)-analysed, written and critically revised jointly by both authors (JLM, PJS).

## Pre-publication history

The pre-publication history for this paper can be accessed here:

http://www.biomedcentral.com/1471-2288/10/32/prepub

## Supplementary Material

Additional file 1 **Reference population-density map of Australia and New Zealand**. Population density map (inhabitants per square kilometre) of Australia (left figure) and New Zealand (right figure); not drawn to scale. Approximate land masses: Australia, 7706143 km^2^; New Zealand 270626 km^2^. Gray-scale legends indicate inhabitant number (range) per km^2^. WA, Western Australia; NY, Northern Territory; QLD, Queensland; NSW, New South Wales; VIC, Victoria; TAS, Tasmania. Click here for file

Additional file 2 **Random effects first-order compartment model**. A brief explanation of the self-starting first-order compartment model. Figure: Parameter estimates with 95%CI for a first-order compartment non-linear (self-starting "ssfol") mixed effects model for geographical descriptors. TAS, Tasmania; NSW, New South Wales; QLD, Queensland; VIC, Victoria; NT, Northern territory; NZ, New Zealand. Click here for file
