# Supplementary material for: Global quantitative indices reflecting provider process-of-care: data-base derivation
Source: BMC Med Res Methodol. 2010 Apr 19;10:32. doi: 10.1186/1471-2288-10-32 (PMC2873511; doi:10.1186/1471-2288-10-32)
Supplement: Additional file 2 — Random effects first-order compartment model. A brief explanation of the self-starting first-order compartment model. Figure: Parameter estimates with 95%CI for a first-order compartment non-linear (self-starting "ssfol") mixed effects model for geographical descriptors. TAS, Tasmania; NSW, New South Wales; QLD, Queensland; VIC, Victoria; NT, Northern territory; NZ, New Zealand. [file 1471-2288-10-32-S2.DOC]

“SSfol” self-starting first-order compartment model

Initial formal analysis utilised non-linear mixed models [28]: in particular, the “SSfol” self-starting first-order compartment model, which is written as:

$y\left(x\right)=\frac{D\text{exp}\left(\theta_{1}\right)\text{exp}\left(\theta_{2}\right)}{\text{exp}\left(\theta_{3}\right)\left[\text{exp}\left(\theta_{2}\right)-\text{exp}\left(\theta_{1}\right)\right]}\left\{\text{exp}\left[-\exp\left(\theta_{1}\right)x\right]-\exp\left[-\exp\left(\theta_{2}\right)x\right]\right\}$, where *D*  is the dose (defined as unity for the purpose of this analysis), $\theta_{1}$ is the logarithm of the elimination rate constant, $\theta_{2}$ is the logarithm of the absorption rate constant and $\theta_{3}$ is the logarithm of the clearance. Log rates and clearances were considered (initially) as random effects, but model parsimony suggested that they should be considered as fixed effects; categorical descriptors were entered (and compared) as random effects. The three fixed model-parameters were significant at *P* < 0.0001.

Parameter estimates and 95% CI for the “ssfol” non-linear mixed effects model for selected descriptors are seen in the Figure below. TAS, Tasmania; NSW, New South Wales; QLD, Queensland; VIC, Victoria; NT, Northern territory; NZ, New Zealand.
