# Supplementary material for: Global quantitative indices reflecting provider process-of-care: data-base derivation
Source: BMC Med Res Methodol. 2010 Apr 19;10:32. doi: 10.1186/1471-2288-10-32 (PMC2873511; doi:10.1186/1471-2288-10-32)
Supplement: Supplementary file 2 [file 1471-2288-10-32-S1.DOC]

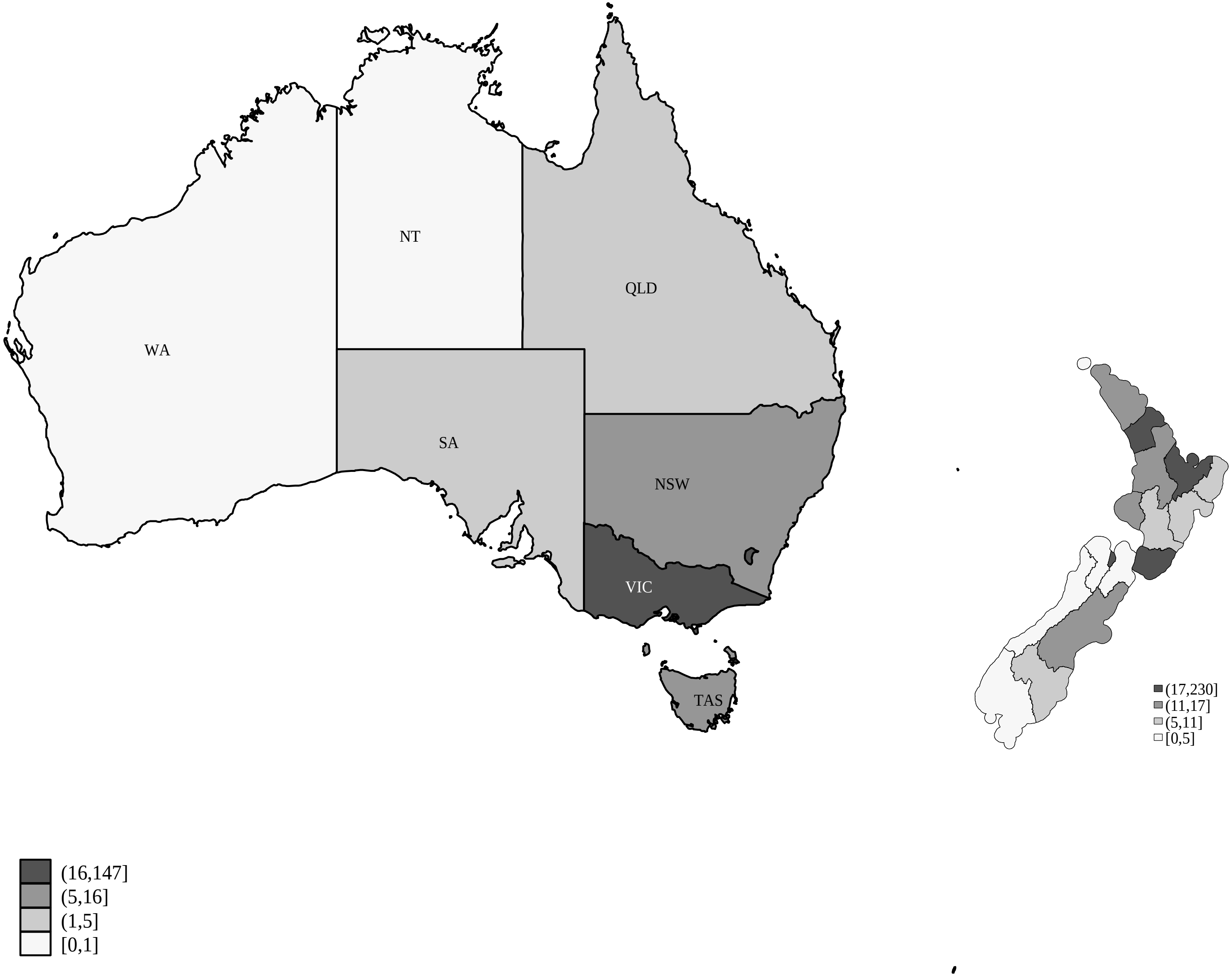
NT
QLD
WA
SA
NSW
VIC
TAS
(17,230]
(11,17]
(5,11]
[0,5]
(16,147]
(5,16]
(1,5]
[0,1]
